# Food Addiction and Stress: Understanding Compulsive Eating in Tunisian Women

**DOI:** 10.1192/j.eurpsy.2026.11370

**Published:** 2026-07-31

**Authors:** K. Mahfoudh, A. Hakiri, A. Jaoua, G. Amri, R. Ghachem

**Affiliations:** Department B, Razi Hospital, Manouba, Tunisia

## Abstract

**Introduction:**

Food addiction, defined as the compulsive consumption of highly palatable foods, is an emerging behavioral health concern. It is associated with overweight, obesity, and psychological distress. Stress has been suggested as a key factor influencing eating behaviors, potentially triggering cravings and compulsive food intake. In Tunisia, social and cultural pressures, lifestyle changes, and exposure to social media may influence women’s dietary habits, yet the prevalence of food addiction and its association with perceived stress remain largely unexplored. Understanding this relationship is crucial for developing effective preventive and supportive interventions.

**Objectives:**

This study aimed to describe the prevalence of food addiction, report Body Mass Index (BMI)) distribution, and examine the association between food addiction and perceived stress levels among Tunisian women.

**Methods:**

A cross-sectional descriptive study was conducted from October 1 to November 30, 2024. Tunisian women aged 18 years and older were recruited online via Facebook, Instagram, and TikTok. Food addiction was assessed using the Arabic validated version of the modified Yale Food Addiction Scale (mYFAS). BMI was calculated from self-reported weight and height and categorized according to WHO criteria (underweight <18.5; normal 18.5–24.9; overweight 25–29.9; obesity ≥30). Perceived stress over the past month was assessed using a five-level Likert scale.

**Results:**

A total of 365 women participated, with a mean age of 33.7 ± 8.9 years (range 18–66). Most participants had a university-level education (90%) and 44.1% lived in the capital. Regarding employment, 56% were employed and 16% were unemployed.

BMI distribution showed that 36.4% had normal weight, 31.8% were overweight, 28.8% obese, and 3.0% underweight. Food addiction was reported by 19.2% of participants. Perceived stress was moderate in 43.8% and high in 31.2% of women. Statistical analysis revealed a significant association between perceived stress and food addiction (p < 0.001), with higher stress levels linked to a greater prevalence of food addiction.

These results indicate that stress may play an important role in the emergence of compulsive eating behaviors.

**Conclusions:**

This study provides descriptive data on food addiction prevalence, BMI distribution, and perceived stress among Tunisian women. The significant association between perceived stress and food addiction highlights the need for preventive strategies and interventions that integrate stress management, nutritional guidance, and psychological support to promote healthier eating behaviors and overall well-being.

**Disclosure of Interest:**

None Declared

